# The intervention effect of internet-based cognitive behavioral therapy on anxiety, depression, and stress in college students: a systematic review and meta-analysis based on randomized controlled trials

**DOI:** 10.3389/fpsyg.2026.1745837

**Published:** 2026-03-09

**Authors:** Jingxia Liu, Yajing Guo, Yuzhu Wu, Nuojia Jin, Yongshu Dong, Xinji Zhao, Xinwang Chen

**Affiliations:** 1 Henan University of Chinese Medicine, Zhengzhou, Henan, China; 2 College of Acupuncture, Moxibustion and Tuina, Henan University of Chinese Medicine, Zhengzhou, Henan, China; 3 Henan Province Integrated Traditional Chinese and Western Medicine Hospital, Zhengzhou, Henan, China

**Keywords:** anxiety, college students, depression, iCBT, meta-analysis

## Abstract

**Background:**

College students face rising anxiety, depression, and stress, with traditional mental health services unable to meet demand. iCBT offers an accessible, low-cost alternative, yet evidence remains inconsistent regarding its effectiveness.

**Objective:**

This systematic review and meta-analysis synthesizes evidence from RCTs to evaluate iCBT’s effects on anxiety, depression, and stress in college students.

**Methods:**

We systematically searched eight databases and one trial registry for studies published up to October 2025. Two reviewers independently screened studies, extracted data, and assessed bias using the Cochrane RoB 2 tool. Inter-rater agreement was measured using Cohen’s kappa coefficient. Meta-analysis and subgroup analyses were conducted using RevMan 5.4 and Stata 17.0.

**Results:**

This systematic review included 30 RCTs involving 5,169 college students, 29 of which were included in the meta-analysis. The meta-analysis results showed that, compared to the control group, internet-based Cognitive Behavioral Therapy significantly alleviated anxiety symptoms (SMD = −0.24, 95% CI −0.31 to −0.18, *p* < 0.001), depressive symptoms (SMD = −0.42, 95% CI −0.54 to −0.30, *p* < 0.001), and stress levels (SMD = −0.37, 95% CI −0.47 to −0.27; *p* < 0.001) in college students. Subgroup analysis tentatively suggested that chatbot-based interventions may be promising for alleviating depression, while web platform-based interventions appeared more effective in improving anxiety. Furthermore, longer intervention durations (>4 weeks) yielded superior effects compared to shorter ones. Follow-up meta-analysis demonstrated that iCBT had a sustained impact on improving college students’ mental health (Depression: SMD = −0.28, 95% CI −0.39 to −0.18, *p* < 0.001; Anxiety: SMD = −0.17, 95% CI −0.30 to −0.03, *p* = 0.01; Stress: SMD = −0.32, 95% CI −0.45 to −0.18, *p* < 0.001).

**Conclusion:**

Our study found that iCBT is an effective approach for improving anxiety, depression, and stress among college students, with relatively long-term effects.

**Systematic review registration:**

https://www.crd.york.ac.uk/PROSPERO/display_record.php?ID=CRD420251177558, identifier (CRD420251177558).

## 1 Introduction

Mental health issues such as anxiety, depression, and stress have become increasingly prominent among college students, emerging as a global public health challenge (Auerbach et al., 2018; Lattie et al., 2019). College life, a period of transition to early adulthood, is accompanied by numerous challenges including academic pressure, social adaptation, financial burdens, and future career development (Karyotaki et al., 2020; Worsley et al., 2021). These factors may lead to psychological distress among students, affecting their academic performance, interpersonal relationships, and overall well-being. Studies have shown that the prevalence of depression among college students is 30.6% (Ibrahim et al., 2013), and mental health problems tend to occur more frequently before the age of 24, highlighting the importance of mental health interventions during the college years (Gambolò et al., 2025). Additionally, the proportion of college students with mental health issues continues to rise, while barriers to accessing traditional mental health treatment persist, such as stigma, limited service accessibility, and time conflicts (Schnyder et al., 2017).

Given the challenges faced by traditional mental health services, internet-based Cognitive Behavioral Therapy (iCBT) has emerged as an accessible and cost-effective alternative, showing great potential in improving mental health (Lattie et al., 2019; Wu et al., 2023). iCBT delivers cognitive behavioral therapy interventions through online platforms, including various forms such as webpages and smartphone applications. Cognitive Behavioral Therapy (CBT) itself is a well-validated psychological treatment method, widely used in addressing various mental disorders such as anxiety and depression (Mason et al., 2023). Its core lies in helping individuals identify and modify negative thought patterns and behavioral habits, thereby improving mood and functioning (Hofmann et al., 2013). With the development of digital technology, CBT has been effectively integrated into digital platforms, giving rise to iCBT. From a theoretical perspective, the appeal of iCBT to college students can be reasonably explained by Self-Determination Theory (SDT) and the Technology Acceptance Model (TAM). SDT emphasizes the important role of autonomy and intrinsic motivation in sustaining engagement (Ryan and Deci, 2020), and the self-guided and flexible nature of iCBT may enhance students’ sense of autonomy and perceived control. TAM posits that perceived usefulness and perceived ease of use are key determinants of technology adoption (Adnan et al., 2025). College students generally demonstrate strong adaptability to online tools, and the anonymity and convenience of iCBT may help reduce help-seeking stigma and practical barriers, thereby improving adherence to the intervention.

Multiple systematic reviews and meta-analyses have confirmed the efficacy of iCBT in treating depression and anxiety (Mamukashvili-Delau et al., 2023; Etzelmueller et al., 2020; Maj et al., 2023). However, evidence synthesis studies on digital mental health interventions for college students still vary in terms of research scope and analytical depth. Oliveira et al. (2023) conducted a meta-analysis focusing on anxiety outcomes among university students, including 15 studies, but did not systematically examine depression or stress, nor did it further explore follow-up effects. Ledur et al. (2023) performed a systematic review that concentrated on the application characteristics of iCBT in college populations (e.g., intervention formats, technical details, and adherence) as well as its preliminary effectiveness; however, the review relied solely on narrative synthesis, without quantitative pooling of effect sizes, and did not analyze potential moderators or long-term follow-up outcomes. Madrid-Cagigal et al. (2025) integrated comprehensive evidence on digital mental health interventions for university students, covering multiple therapeutic approaches (CBT, ACT, mindfulness) and various digital delivery formats. Although the study reported overall moderate effect sizes for depression and anxiety, it did not provide a separate analysis of iCBT as a specific intervention type, nor did it conduct an independent quantitative synthesis of stress outcomes. In addition, long-term follow-up effects were not systematically analyzed, making it difficult to fully elucidate the context-specific effects of iCBT among college students and its potential moderating mechanisms.

In addition, findings from existing randomized controlled trials (RCTs) remain heterogeneous, primarily reflected in differences in digital delivery formats (e.g., app-based vs. web-based platforms), variation in intervention duration, and inconsistencies in the maintenance of effects at follow-up. As a key target group for iCBT interventions, college students’ mental health is directly related to their academic success and social adaptation. Therefore, it is necessary to conduct an updated meta-analysis focusing on iCBT. This study will explore potential moderators through subgroup analyses, synthesize intervention effects at the follow-up stage to assess long-term efficacy, and systematically evaluate three core outcomes (anxiety, depression, and stress), thereby providing more targeted and reliable evidence-based insights.

This systematic review and meta-analysis aims to synthesize evidence from existing RCTs to evaluate the impact of iCBT on anxiety, depression, and stress among college students. We will systematically review and analyze relevant studies to assess the effectiveness of iCBT in alleviating anxiety, depression, and stress symptoms in this population, as well as identify factors that may moderate the intervention effects. Through the systematic integration of this evidence, this study will provide an evidence-based foundation for mental health services in colleges and universities, guide the design and implementation of future iCBT programs, and offer references for formulating strategies to promote college students’ mental health.

## 2 Methods

This study was conducted in accordance with the PRISMA (Preferred Reporting Items for Systematic Reviews and Meta-Analyses) guidelines (Moher et al., 2009) (Supplementary Appendix 1). The systematic review protocol was registered in the International Prospective Register of Systematic Reviews (PROSPERO, registration number: CRD420251177558).

### 2.1 Search strategy

After finalizing the search strategy in consultation with information specialists, we conducted a systematic search across eight English databases (PubMed, Web of Science, Cochrane Library, Embase, PsycINFO, Scopus, CINAHL, and PsycARTICLES) and one clinical trial registry platform.^1^ The search period spanned from the establishment of each database to October 12, 2025, with the search language restricted to English. The search strategy combined subject terms and free-text words, including key term combinations such as college students (university student, college student, undergraduate*, etc.), cognitive behavioral therapy (cognitive behavioral therapy, CBT, etc.), internet-based (mobile*, internet*, web, eHealth, digital, etc.), and randomized controlled trials (randomized controlled trial, RCT, etc.), which were combined using Boolean operators. The detailed search strategy is provided in Supplementary Appendix 2.

### 2.2 Inclusion and exclusion criteria

#### 2.2.1 Inclusion criteria

The inclusion criteria were developed according to the PICOS framework, as detailed below:

##### 2.2.1.1 Population

The study population consisted of college students (including undergraduate, graduate, and other students in higher education institutions), regardless of gender, age, major, or nationality.

##### 2.2.1.2 Intervention

Internet-based Cognitive Behavioral Therapy, specifically interventions delivered via the internet, mobile applications, or digital platforms.

##### 2.2.1.3 Comparison

The control condition consisted of any type of non-iCBT intervention control group (e.g., waitlist control, treatment-as-usual, placebo intervention group, or other non-iCBT control groups).

##### 2.2.1.4 Outcomes

Studies that included anxiety (e.g., generalized anxiety, state–trait anxiety assessed by scales such as GAD-7, STAI, HADS-A, and DASS), depression, or stress as outcome measures.

##### 2.2.1.5 Study design

Only RCTs were included.

#### 2.2.2 Exclusion criteria

Studies were excluded if any of the following conditions applied: (1) The full text was unavailable after contacting the authors, and crucial data could not be extracted; (2) The outcome measures were unclear, and the data could not be merged or converted for analysis; (3) The study was a duplicate publication; (4) The study was exploratory in nature; (5) The publication language was non-English.

### 2.3 Study selection and data extraction

All retrieved studies were imported into the EndNote 9 (Clarivate Analytics) reference management software. Two reviewers (JXL and YZW) independently screened the titles and abstracts for eligibility based on the inclusion and exclusion criteria. The full texts of potentially relevant studies were reviewed to select those meeting the criteria, and reasons for exclusion were recorded. Disagreements were resolved through discussion, with a third author (YJG) adjudicating when necessary. The inter-rater agreement was measured using Cohen’s kappa coefficient (McHugh, 2012). The extracted data included: source information (authors, publication year, and country), sample characteristics (sample size, mean age, proportion of males), intervention details (type, duration), measurement methods (scales), and outcome measures (anxiety, depression, and stress). For key data missing from the articles, the research team contacted the corresponding authors via email to obtain supplementary information. Changes in the mean scores and standard deviations of depression, anxiety, and stress scale scores from pre-intervention (baseline) to post-intervention and at follow-up were extracted. The SD change (SD difference) was calculated using formulas from the Cochrane Handbook (Higgins et al., 2019). If a study reported outcome data at multiple follow-up time points, data from the longest follow-up period for which all relevant study groups (intervention and control groups) reported outcomes were extracted (Cochrane Handbook, section 9.3.4) (Higgins and Green, 2011). Furthermore, all extracted data were verified by a third reviewer (YJG) to ensure accuracy and reliability.

### 2.4 Risk-of-bias assessment

The quality of each included study was evaluated using the guidelines provided in the Cochrane Risk of Bias Tool 2.0 (RoB 2) (Sterne et al., 2019), which assesses the risk of bias across five domains: randomization bias, allocation bias, attrition bias, outcome measurement bias, and selective reporting bias. Each domain was rated as “low risk of bias,” “some concerns,” or “high risk of bias.” A study was classified as “low overall risk” if all domains were rated “low risk of bias,” and as “high overall risk” if at least one domain was rated “high risk of bias.” This assessment was independently conducted by two reviewers (JXL and YJG), with disagreements resolved through discussion.

### 2.5 Data synthesis and meta-analysis

Data analysis was performed using RevMan 5.4 (Cochrane Collaboration) and Stata 17.0 software. Due to the use of different measurement indicators across studies, the standardized mean difference (SMD) was adopted for comprehensive evaluation in this meta-analysis. The degree of heterogeneity among studies was assessed using the *I^2^* statistic (I-squared), which was categorized as low heterogeneity (0% ≤ *I^2^* ≤ 25%), moderate heterogeneity (26% ≤ *I^2^* ≤ 50%), and high heterogeneity (*I^2^* > 50%) (Higgins et al., 2003). A random-effects model was used when significant heterogeneity existed (*I^2^* > 50%), while a fixed-effects model was applied if heterogeneity was low (*I^2^* < 50%). Leave-one-out sensitivity analysis was conducted using RevMan 5.4, where individual studies were sequentially excluded to analyze their impact on the overall results and heterogeneity. If the overall heterogeneity significantly decreased after removing a single study, that study was considered a potential source of heterogeneity. Additional sensitivity analyses were performed using Stata 17.0.

Subgroup analyses based on intervention type (app-based, web platform-based, and chatbot-based) and intervention duration (≤4 weeks, >4 and ≤8 weeks, >8 weeks) were conducted using RevMan 5.4. These analyses aimed to explore the differences in effectiveness across various intervention conditions and durations.

Publication bias was assessed using quantitative Egger’s test and visual inspection of funnel plot asymmetry. When the number of included studies exceeded 10, we utilized both the graphical symmetry of funnel plots and the Egger’s test statistic to detect potential publication bias, as the statistical power of these tests is reduced with a smaller number of studies (Higgins et al., 2019; Sterne et al., 2011). The absence of significant publication bias was concluded if the funnel plot exhibited a symmetric distribution and the *p*-value from Egger’s test was greater than 0.05 (Zwetsloot et al., 2017).

## 3 Results

### 3.1 Literature selection

**Figure 1:**
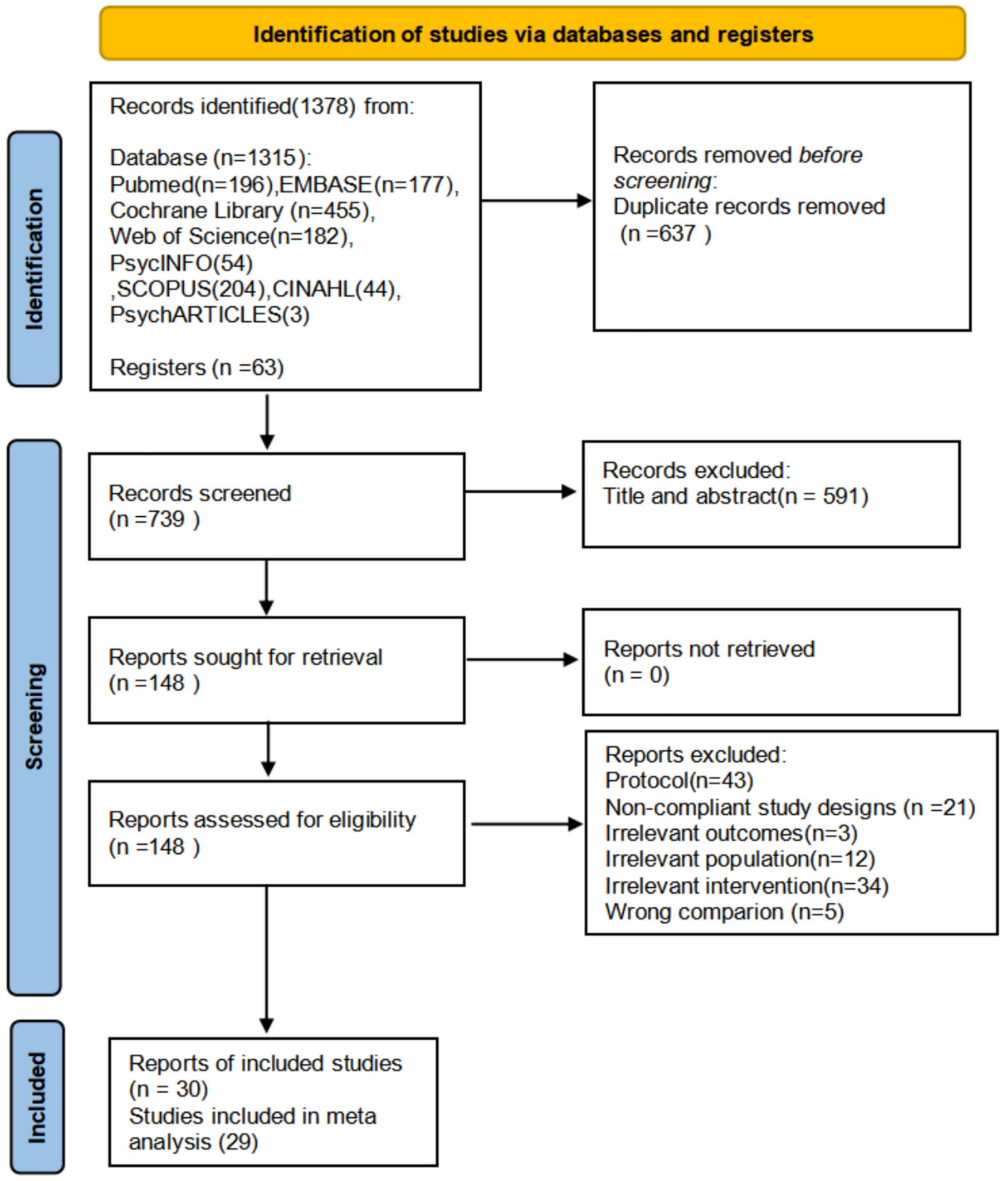
Flowchart of PRISMA (preferred reporting items for systematic evaluation and meta-analysis).

Our search initially identified a total of 1,378 citations, which were imported into EndNote X9. After removing 637 duplicate records, 739 articles remained for title and abstract screening. Following the title and abstract screening, 148 full-text articles were assessed for eligibility. Cohen’s *κ* coefficient for inter-rater agreement at this stage was 0.79, which indicated substantial agreement between the two independent reviewers. Of these, 118 articles (80%) were excluded, and 30 studies (20%) met the inclusion criteria for the systematic review. Among the 30 studies eligible for the systematic review, 29 provided complete data suitable for inclusion in at least one meta-analysis. Specifically, 26 studies (89.7%) reported sufficient data for the meta-analysis of depressive symptoms, 20 studies (69%) reported sufficient data for the meta-analysis of anxiety symptoms, and 9 studies (31%) reported sufficient data for the meta-analysis of stress symptoms (Figure 1).

### 3.2 Study characteristics

Table 1 summarizes the basic characteristics of the 30 included studies. A total of 5,169 participants were enrolled in this analysis, with the sample size of each study ranging from 19 to 874. These 30 studies were conducted across 15 countries spanning five continents, with Asia accounting for 36.7% (11/30), Europe 23.3% (7/30), North America 30% (9/30), Oceania 6.7% (2/30), and South America 3.3% (1/30). Among these RCTs, 27 (90%) reported depression outcomes, 20 (66.7%) reported anxiety outcomes, and 9 (30%) reported stress outcomes. Regarding intervention delivery modes, 8 studies (26.7%) adopted app-based protocols, 19 (63.3%) used web-based platforms, and 3 (10%) employed AI chatbots.

**Table 1:** Included study characteristics.

Study	Country	Intervention/control		Intervention	Control	Duration	Outcomes	Measurement	Results of the study	Male proportion	dropout rate (%)
		Sample size, N	Mean age mean (SD), y								I/C
Abramovitch et al., (2024)	United States	35/35(70)	18.78(0.97)	Gamified application based on CBT (GG OCD app)	WLC	2 weeks;1 month after the intervention follow-up	a. Depression b. Anxiety c. Stress	DASS-21 DASS-21 DASS-21	a,b: No significant difference c: I < C	14.30%	11.4/11.4
Amarnath et al. (2025)	Netherlands	199/204(403)	23.46(4.62)	uided internet-based intervention (GetStarted) based on the principles of CBT	WLC	4 weeks; Only the intervention group was followed up at 6 months after the intervention	a. Depression b. Anxiety c. Stress	PHQ - 9 GAD - 7 PSS-10	a: I < C b: I < C c: I < C	29.53%	50.3/34.3
Cook et al. (2019)	United Kingdom	82/77(159)	20.43 (1.65)/20.27 (1.55)	i-RFCBT	TAU	10 weeks; no follow-up	a. Depression b. Anxiety	PHQ - 9 GAD - 7	a: I < C b: I < C	20.48%	28/14.3
Day et al. (2013)	Canada	33/33(66)	23.55 (4.98)	Online self-help program based on CBT	WLC	6 weeks; no follow-up	a. Depression b. Anxiety c. Stress	DASS-21 DASS-21 DASS-21	a: I < C b: I < C c: I < C	10.61%	12.1/27.3
Fitzpatrick et al. (2017)	United States	34/36(70)	22.2(2.33)	Automated Conversational Agent (Woebot) Based on CBT	Information control	2 weeks; no follow-up	a. Depression b. Anxiety	PHQ - 9 GAD - 7	a: I < C b: I < C	14%	9/31
Floyd and Vargas (2025)	United States	27/28(55)	19.9(0.97)	CBT-I Coach	WLC	4 weeks; no follow-up	a. Depression	CES-D	a: I < C	18.20%	11.1/21.4
Hamid et al. (2022)	Pakistan	20/20(40)	18–25	CBT app	WLC	2 weeks; no follow-up	a. Depression b. Anxiety	PHQ - 9 GAD - 7	a: I < C b: I < C	53.30%	0/0
Howell et al. (2019)	United States	266/328(594)	28.0(8.1)/(27.61)	Web CBT	no treat ment	4 weeks; no follow-up	b. Anxiety	GAD - 7	b: I < C	32%	24.8/13.1
Karyotaki et al. (2022)	Netherlands	48/52(100)	21.91 (2.61)	iCBT	TAU	7 weeks;6 and 12 months post-randomization follow-up	a. Depression b. Anxiety	PHQ - 9 GAD - 7	No significant difference	28.75%	27.1/21.2
Kim et al. (2025)	Republic of Korea	91/79(170)	22.60 (3.37)	CBT app	WLC	30 days;2 months after the intervention follow-up	a. Depression	PHQ - 9	a: I < C	20%	14.3/1.3
Kim and Lee (2023)	Republic of Korea	18/16(34)	19–29	Roy Adaptation Model (RAM)	WLC	1 month1 months after the intervention follow-up	a. Depression	CES - D	a: I < C	44.58%	14.3/15.8
Lambert et al. (2025)	United Kingdom	203/204(407)	22.49 (7.28)	Web-Based Common Elements Toolbox (COMET) Single-Session Interventions	WLC	60 - 75 M inutes;4 weeks after the intervention follow-up	a. Depression b. Anxiety c. Stress	PHQ-9 GAD-7 PSS-4	a: I < C b: No significant difference c: I < C	16.30%	11.3/6.99
Lee et al. (2025)	Republic of Korea	47/38(85)	20.6(2.0)/20.2(1.9)	Moa chatbot(CBT)	regular functions	1 month; no follow-up	c. Stress	PSS	c: I < C	56.47%	12.8/10.5
Lin et al. (2025)	China	46/45(91)	21.44(0.96)	CBT-based mobile application	TAU	12 weeks; no follow-up	a. Depression	TDICS	No significant difference	15.40%	0/0
Liu et al. (2022)	China	41/42(83)	23.08(1.76)	AI chatbots(Constructed based on the principles of Cognitive Behavioral Therapy)	a bibliotherapy control group	16 weeks; no follow-up	a. Depression b. Anxiety	PHQ - 9 GAD - 7	a: I < C b: No significant difference	44.58%	19.5/28.6
McCloud et al. (2020)	United Kingdom	84/84(168)	25.1 (7.68)	CBT app	WLC	6 weeks; no follow-up	a. Depression b. Anxiety	HADS-D HADS-A	Depression: No significant difference	13.69%	51.2/32.1
McDermott and Dozois (2019)	Canada	144/158(302)	18.82(1.77)/18.75(1.63)	MoodGYM(iCBT)	Attentional Control	6 weeks;4 months after the intervention follow-up	a. Depression	DASS-12	a: I < C	28.48%	26.4/20.3
Melnyk et al. (2015)	United States	82/39(121)	18.8(4.8)/18.4(1.9)	COPE (Online Cognitive-Behavioral Skill-Building Program)	standard freshman curriculum	10-12 weeks; no follow-up	a. Depression b. Anxiety	PHQ - 9 GAD - 7	No significant difference	15.7%	25.6/17.9
Mohammadi et al. (2025)	Iran	23/23(46)	27.85(4.94)	CBT app	CBT	10 weeks;3 months after the intervention follow-up	a. Depression b. Anxiety c. Stress	DASS-42 DASS-42 DASS-42	a: I < C b: I < C c: I < C	26.09%	11.5/11.5
Morris et al. (2016)	United Kingdom	43/47(90)	20.5 (1.95)	iCBT	WLC	6 weeks; no follow-up	a. Depression b. Anxiety	BDI-II STAI-S	a: I < C b: I < C	28.89%	25.6/6.4
Mullin et al. (2015)	Australia	30/23(53)	28.6(10.05)/ 26.9(11.51)	UniWellbeing Course (iCBT)	WLC	6 weeks; no follow-up	a. Depression b. Anxiety	PHQ - 9 GAD - 7	a: I < C b: I < C	35.80%	30/8.7
Newman et al. (2021)	India	117/105(222)	19.9(1.56)	Internet-Delivered Guided Self-Help	WLC	3 months; no follow-up	a. Depression b. Anxiety	DASS GAD-Q-IV	a: I < C b: I < C	68.90%	59/61
Newman et al. (2021)	United States	50/50(100)	21.62/21.18	CBT app	no treat ment	3 months;6 months after the intervention follow-up	c. Stress	DASS-21	c: I < C	23.00%	22/14
Okajima et al. (2022)	Japan	24/24(48)	19.56 (1.86)	REFRESH (E-mail-Delivered CBT-I)	Self-Monitoring	8 weeks; no follow-up	a. Depression b. Anxiety c. Stress	DASS-21 DASS-21 DASS-21	a: I < C b: I < C c: I < C	33%	12.5/16.7
Richards et al. (2016)	Ireland	70/67(137)	23.82(7.05)	iCBT	WLC	6 weeks; no follow-up	a. Depression b. Anxiety	BDI-II GAD - 7	a: I < C b: I < C	22.63%	15.7/20.9
Salamanca-Sanabria et al. (2020)	Colombia	107/107(214)	22.15 (4.74)	iCBT	WLC	7 weeks; Only the intervention group was followed up at 3 months after the intervention	a. Depression	PHQ - 9	a: I < C b: I < C	28.50%	80.4/49.6
Sethi et al. (2010)	Australia	9/10(19)	19.47(1.57)	MoodGYM(online CBT)	no treat ment	3 weeks; no follow-up	a. Depression b. Anxiety	DASS-21 DASS-21	a: No significant difference b: I < C	21.05%	0/0
Toh et al. (2022)	Singapore	135/129(264)	22.5(5.41)	APP (CBT)	active control group	8 days;1 month after the intervention follow-up	a. Depression b. Anxiety c. Stress	PHQ - 9 GAD - 7 PSM-9	a: No significant difference b: I < C c: I < C	25.00%	16.7/18.9
Xu and Ma (2025)	China	42/42(84)	23.3(1.07)	AI chatbots (CBT)	Provide a low-social-cue AI chatbot with text-only functionality	16 weeks; no follow-up	a. Depression	PHQ - 9	a: I < C	51.19%	14.3/23.8
Benjet et al. (2023)	Mexico	439/435(874)	21.4 (3.2)	i-CBT	TAU	8 weeks;3 months post-randomization follow-up	a. Depression b. Anxiety	PHQ - 9 GAD - 7	a: I < C b: I < C	21.51%	37.8/23

a, Depression; b, Anxiety; c, Stress; I, Intervention; C, Control; PHQ – 9, Patient Health Questionnaire- 9; GAD – 7, Generalized Anxiety Disorder-7; DASS-21, Depression, Anxiety and Stress Scale-21; HADS-A, Hospital Anxiety and Depression Scale-Anxiety Subscale; DASS-42, Depression, Anxiety and Stress Scale-42; TDICS, Tung’s Depression Inventory for College Students; STAI, State Trait Anxiety Inventory; GAD-Q-IV, Generalized Anxiety Disorder Questionnaire, Fourth Edition; PSS, Perceived Stress Scale; PSM-9, Psychological Stress Measure.

### 3.3 Scales

This study employed various scales to assess symptoms of anxiety, depression, and stress. For the evaluation of anxiety symptoms across 21 studies, the GAD-7 was the most widely used tool, applied in 13 studies (61.9%), followed by the DASS, which was used in 5 studies (23.8%). In the assessment of depressive symptoms across 27 studies, the PHQ-9 was the primary instrument, utilized in 12 studies (44.4%), and the depression subscale of the Depression, Anxiety, and Stress Scales was also commonly used, being featured in 9 studies (33.3%). For the evaluation of stress levels across 9 studies, the DASS was used in 5 studies (55.6%), the PSS in 3 studies (33.3%), and the PSM-9 in 1 study (11.1%).

### 3.4 Intervention duration and control group categories

In the included studies, there was variability in both intervention duration and the types of control conditions. Regarding control group type, no-treatment controls were the most common, implemented in 17 studies (56.7%), including waitlist and no-treatment control groups. Treatment as usual was used in 4 studies (13.3%). Active control conditions were implemented in 9 studies (30%), including information control, attention control, minimal intervention, self-monitoring, bibliotherapy and low-social-cue AI chatbot interventions. In terms of intervention duration, 9 studies (30.0%) had a duration of ≤4 weeks, 13 studies (43.3%) lasted between >4 and ≤8 weeks, and 8 studies (26.7%) extended beyond 8 weeks.

### 3.5 Risk of bias

**Figure 2:**
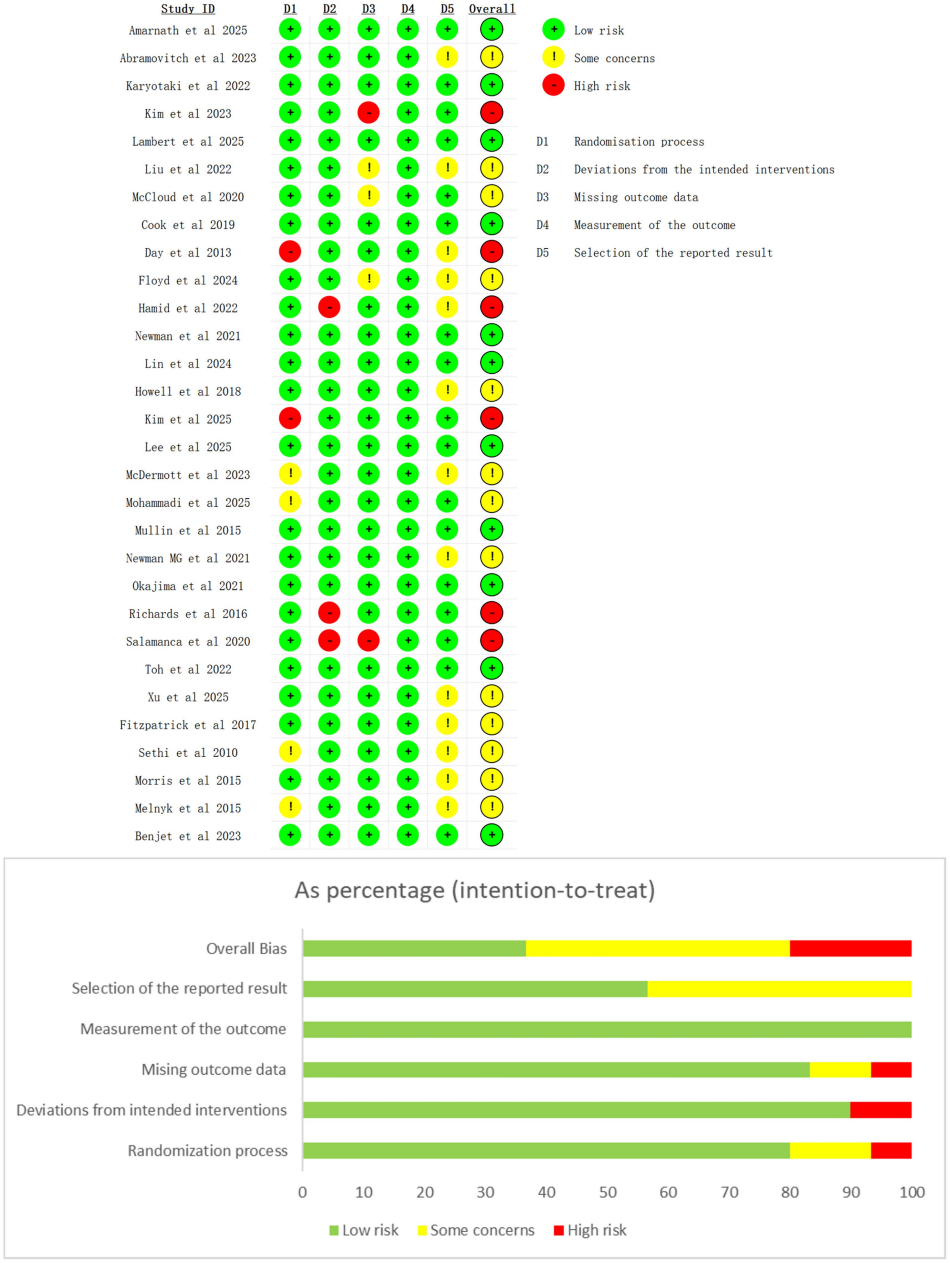
Risk of bias summary and chart.

All included studies were assessed for risk of bias across the following domains: randomization process, allocation concealment, blinding of participants and researchers, blinding of outcome assessment, outcome measures, bias due to missing outcome data, selective reporting, and other biases. The results (Figure 2) showed that 36.7% (11/30) of the studies were rated as low risk, 43.3% (13/30) as having some concerns, and 20% (6/30) as high risk. Among the 13 studies with some concerns (i.e., at least one domain was rated as “some concerns”), the main issues included: no mention of allocation concealment (McDermott and Dozois, 2019; Melnyk et al., 2015; Mohammadi et al., 2025; Sethi et al., 2010), missing outcome data (Floyd and Vargas, 2025; Liu et al., 2022; McCloud et al., 2020), and lack of reporting of study protocols or trial registration information (Abramovitch et al., 2024; Day et al., 2013; Fitzpatrick et al., 2017; Floyd and Vargas, 2025; Hamid et al., 2022; Howell et al., 2019; Liu et al., 2022; McDermott and Dozois, 2019; Melnyk et al., 2015; Morris et al., 2016; Newman et al., 2021; Sethi et al., 2010; Xu and Ma, 2025). For the 6 high-risk studies, 2 studies (Day et al., 2013; Kim et al., 2025) lacked allocation concealment; 3 studies (Hamid et al., 2022; Richards et al., 2016; Salamanca-Sanabria et al., 2020) had deviations from the predefined intervention protocol; and 2 studies (Kim and Lee, 2023; Salamanca-Sanabria et al., 2020) had group differences in missing data.

## 4 Meta-analysis findings

### 4.1 Depression

**Figure 3:**
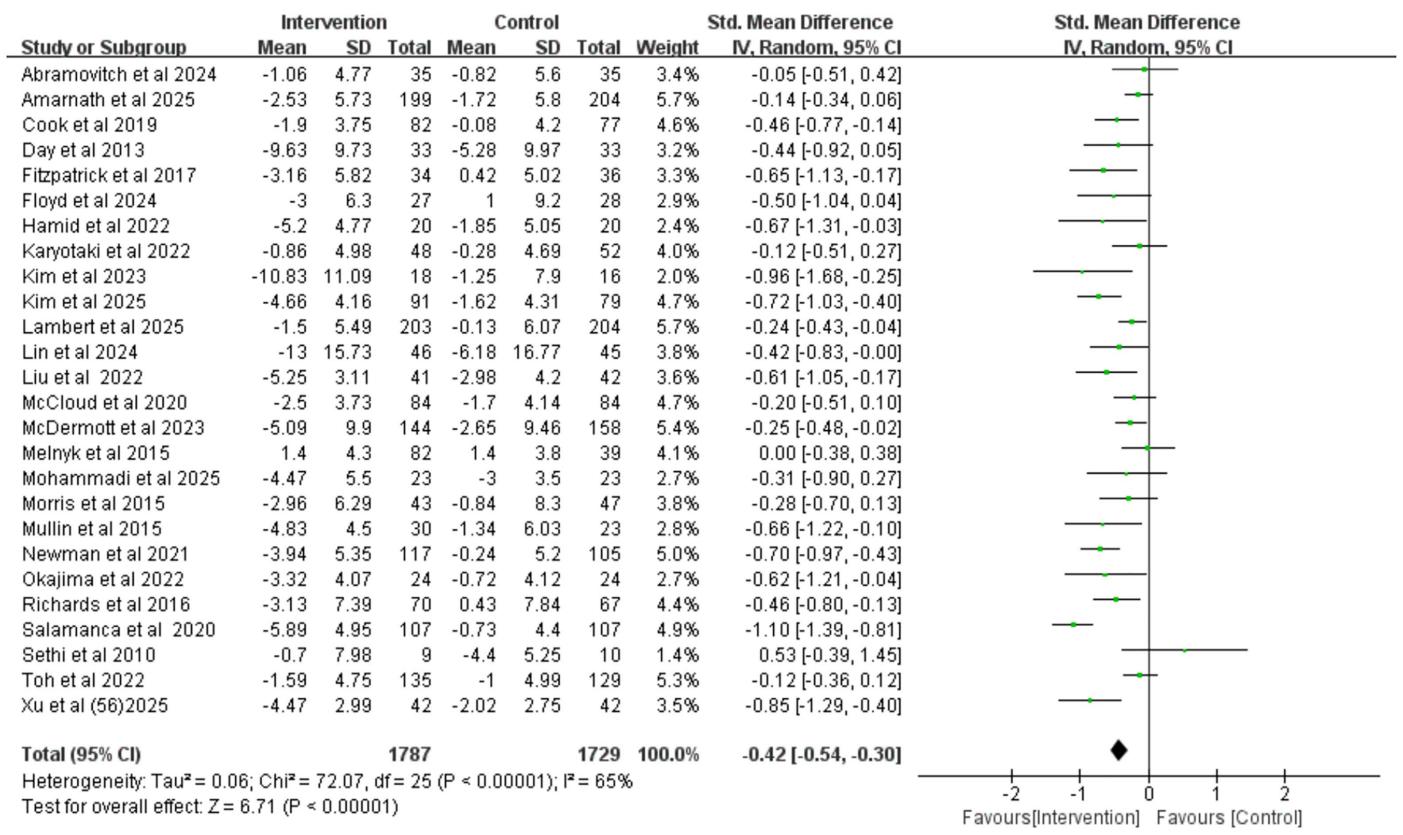
Forest plot of iCBT on depression.

The pooled results of 26 randomized controlled trials (involving a total of 3,516 participants) showed (Figure 3) that, compared with the control group, the iCBT intervention group achieved a small-to-moderate yet significant improvement in depression (SMD = −0.42, 95% CI −0.54 to −0.30, *p* < 0.001). High heterogeneity was observed among the studies (*I^2^* = 65%, *p* < 0.001). Egger’s test (*p* = 0.237) and visual inspection of funnel plot symmetry (Supplementary Figure S1) indicated no significant publication.

**Figure 4:**
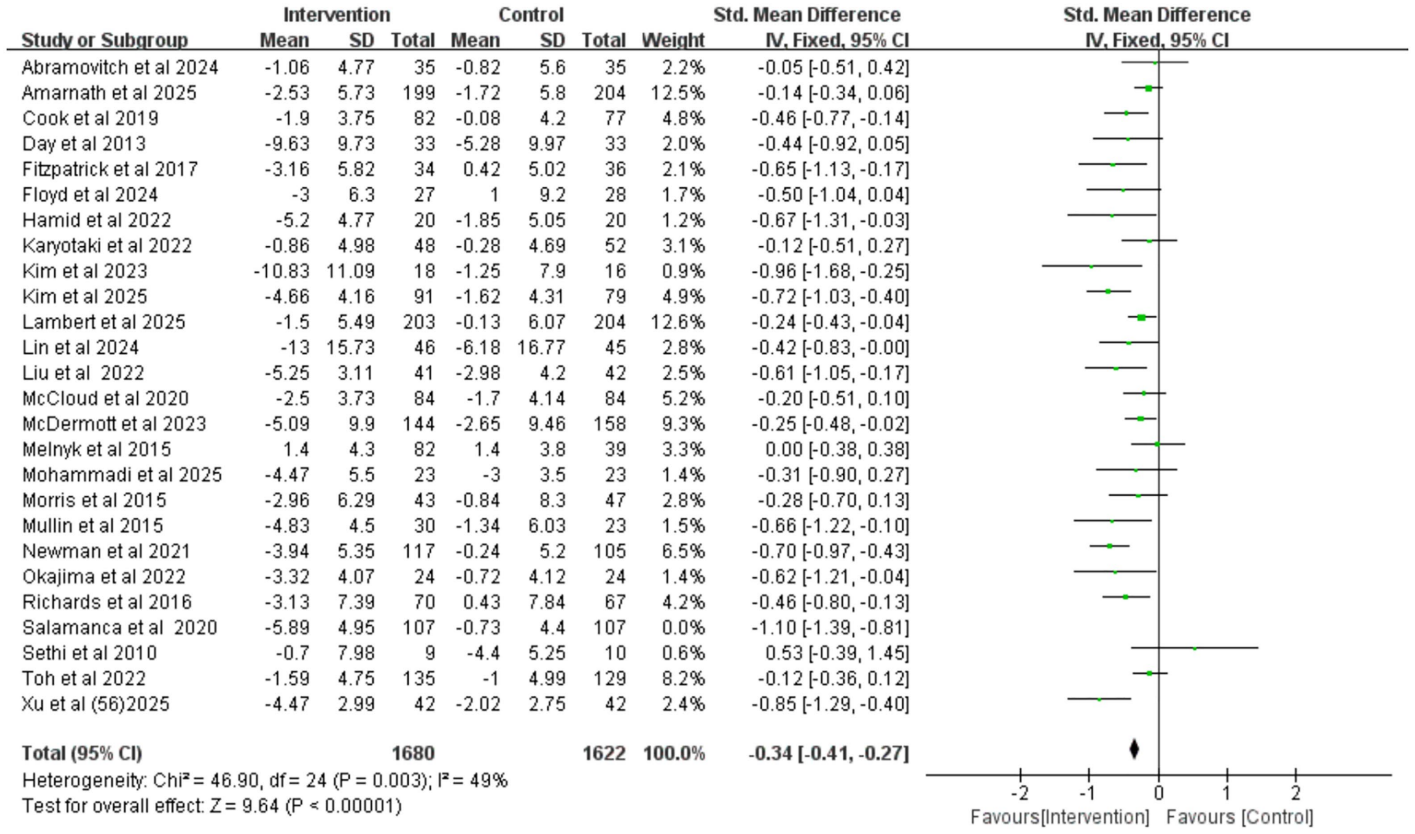
Forest plot for depression after study exclusion.

**Figure 5:**
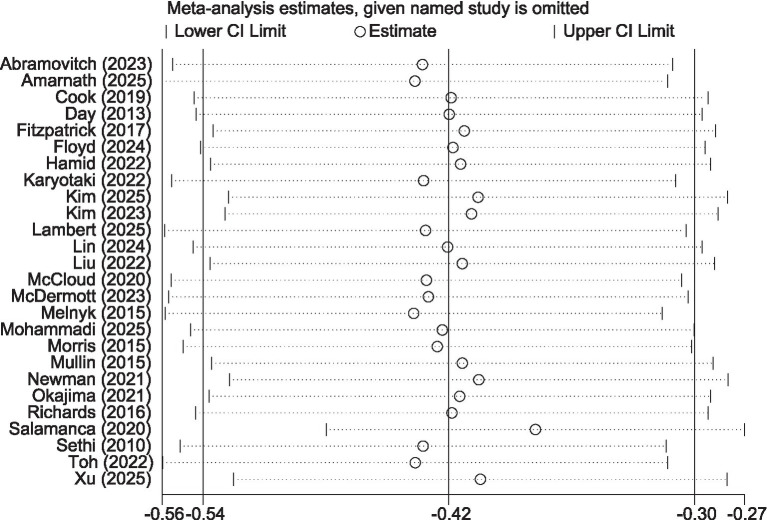
Sensitivity analysis plot for depression.

After excluding the study by Salamanca-Sanabria et al. (2020), which was rated as high risk of bias due to inadequate allocation concealment and missing outcome data, the heterogeneity among the remaining studies was reduced to a moderate level (*I^2^* = 49%, *p* = 0.003), indicating that this study was a potential source of high heterogeneity. A subsequent meta-analysis using a fixed-effects model (Figure 4) still yielded a significant result (SMD = −0.34, 95% CI −0.41 to −0.27, *p* < 0.001), demonstrating the robustness of iCBT’s effect in improving depression among college students. This conclusion was further supported by additional sensitivity analyses (Figure 5).

### 4.2 Anxiety

**Figure 6:**
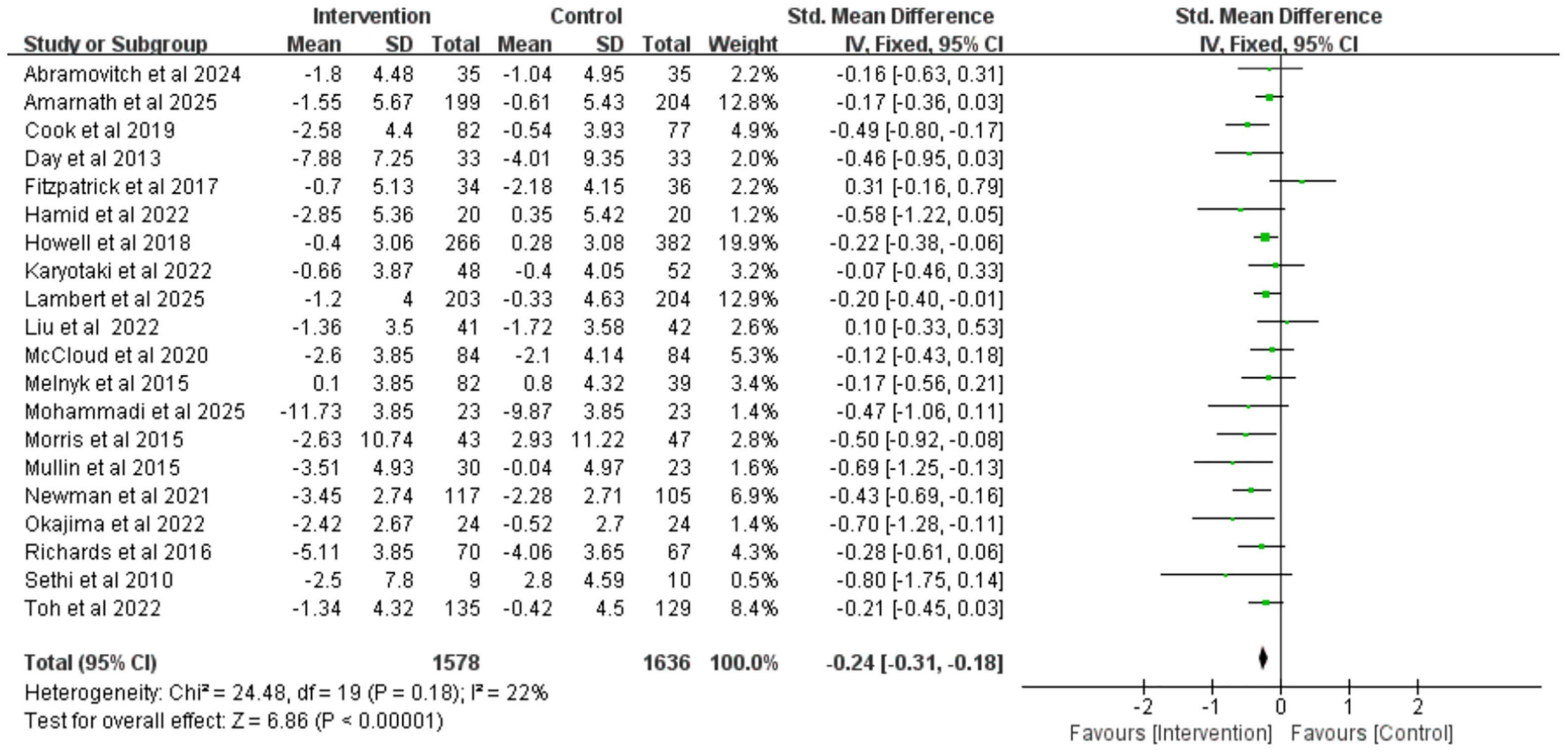
Forest plot of iCBT on anxiety.

**Figure 7:**
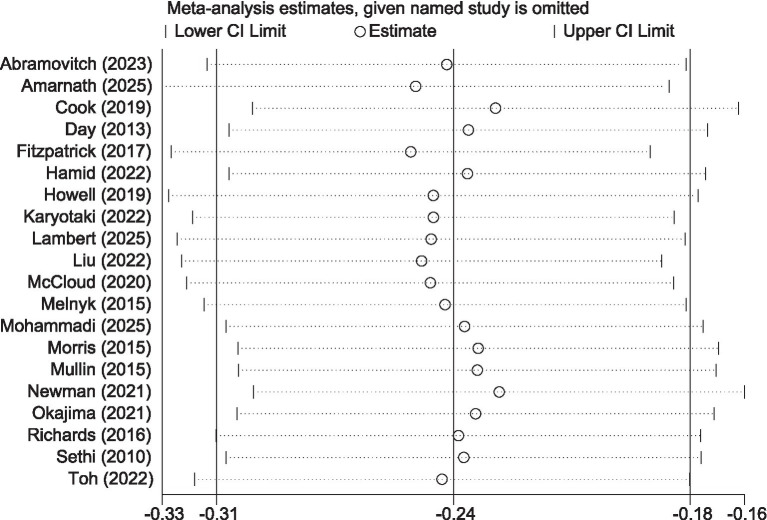
Sensitivity analysis plot for anxiety outcomes.

A meta-analysis was performed on anxiety data from 20 studies (involving a total of 3,214 college students). Given the low heterogeneity among the studies (*I^2^* = 22%, *p* = 0.18), a fixed-effects model was used. The results (Figure 6) showed that college students receiving iCBT intervention had significantly lower anxiety symptoms compared to the control group (SMD = −0.24, 95% CI -0.31 to −0.18, *p* < 0.001). Although the effect size was small, the effect was clear. Publication bias assessment indicated no significant bias, as shown by Egger’s test (*p* = 0.166) and funnel plot symmetry (Supplementary Figure S2). Leave-one-out sensitivity analysis further supported the stability of the analytical conclusions (Figure 7).

### 4.3 Stress

**Figure 8:**
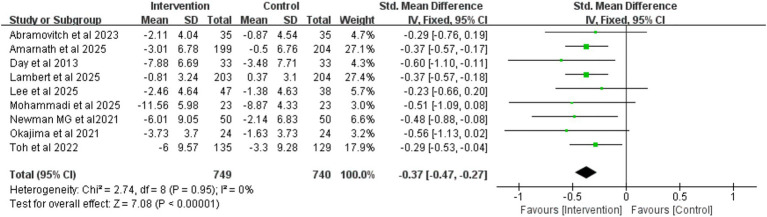
Forest plot of iCBT on stress.

**Figure 9:**
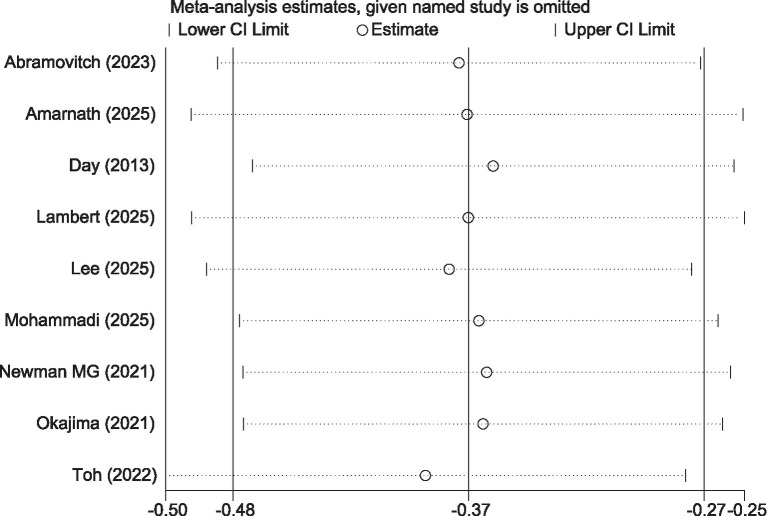
Sensitivity analysis plot for stress.

Stress data from 9 studies (involving a total of 1,489 participants) were analyzed. Given the extremely low heterogeneity among the studies (*I^2^* = 0%, *p* = 0.95), a fixed-effects model was employed. The meta-analysis results (Figure 8) showed that, compared with the control group, the iCBT intervention group achieved a small yet significant improvement in stress reduction (SMD = −0.37, 95% CI −0.47 to −0.27, *p* < 0.001). Furthermore, the results of the sensitivity analysis (Figure 9) supported the reliability of the pooled findings.

## 5 Subgroup analysis results

### 5.1 Intervention type

Results of the subgroup analysis by intervention type (Supplementary Appendix 3) indicated differences in the effects of various iCBT formats on anxiety and depression. Due to the limited number of studies evaluating stress outcomes, no subgroup analysis by intervention type was conducted for this indicator. When stratified into three categories based on intervention format (app-based, web platform-based, and chatbot-based), the analysis showed the following: For depression outcomes (Supplementary Figure S3), the effect size of app-based interventions was (SMD = −0.35, 95% CI − 0.54 to −0.16), the effect size of web platform–based interventions was (SMD = −0.41, 95% CI − 0.58 to −0.23), and the effect size of chatbot-based interventions was (SMD = −0.70, 95% CI − 0.97 to −0.44). No statistically significant differences were observed between subgroups (*p* = 0.08). For anxiety outcomes (Supplementary Figure S4), the effect size of app-based interventions was (SMD = −0.22, 95% CI − 0.39 to −0.06), the effect size of web platform–based interventions was (SMD = −0.28, 95% CI − 0.36 to −0.20), and the effect size of chatbot-based interventions was (SMD = 0.20, 95% CI − 0.12 to 0.52). The differences between subgroups were statistically significant (*p* = 0.02).

### 5.2 Intervention duration

Due to the limited number of studies reporting stress outcomes, no subgroup analysis based on intervention duration was conducted for this indicator. Intervention duration was categorized into three groups (≤4 weeks, >4 to ≤8 weeks, and >8 weeks). The results showed that for depression outcomes (Supplementary Figure S5), the effect size for interventions lasting ≤4 weeks was (SMD = −0.23, 95% CI − 0.37 to −0.08), the effect size for interventions lasting >4 and ≤8 weeks was (SMD = −0.51, 95% CI − 0.72 to −0.30), and the effect size for interventions lasting >8 weeks was (SMD = −0.49, 95% CI − 0.69 to −0.28). The differences between subgroups were statistically significant (*p* = 0.04). For anxiety outcomes (Supplementary Figure S6), the pooled effect size for interventions lasting ≤4 weeks was (SMD = −0.19, 95% CI − 0.28 to −0.10), the pooled effect size for interventions lasting >4 and ≤8 weeks was (SMD = −0.31, 95% CI − 0.47 to −0.16), and the pooled effect size for interventions lasting >8 weeks was (SMD = −0.33, 95% CI − 0.49 to −0.17). No statistically significant differences were observed between subgroups (*p* = 0.22).

## 6 Follow-up results

**Figure 10:**
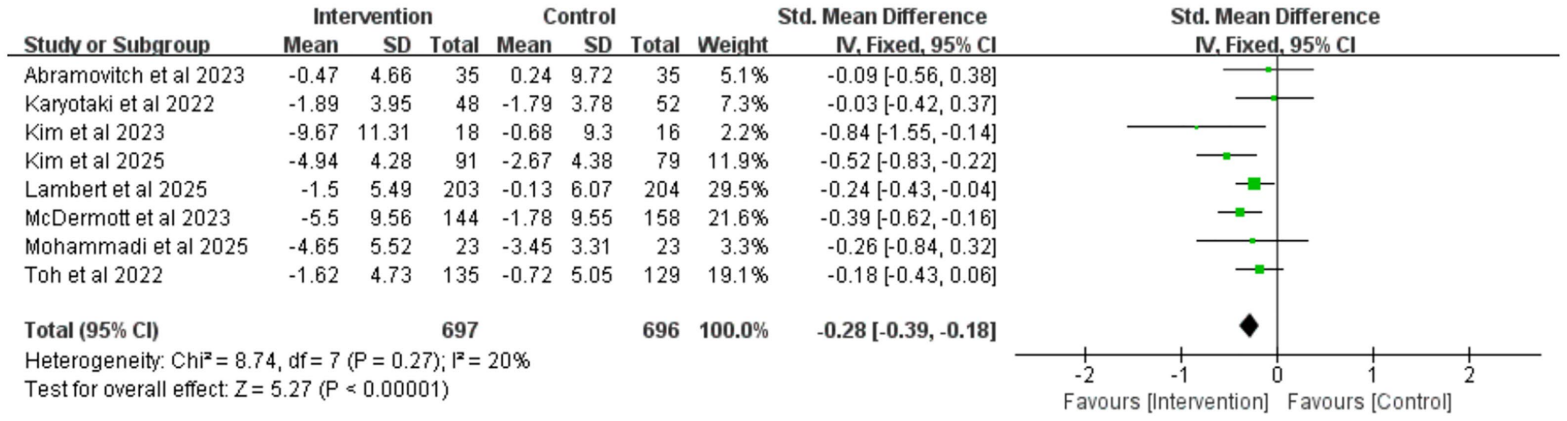
Meta-analysis and forest plot of depression at follow-up.

**Figure 11:**
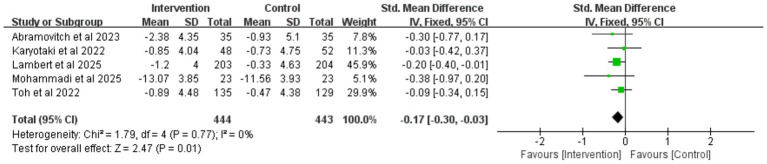
Meta-analysis and forest plot of anxiety at follow-up.

**Figure 12:**
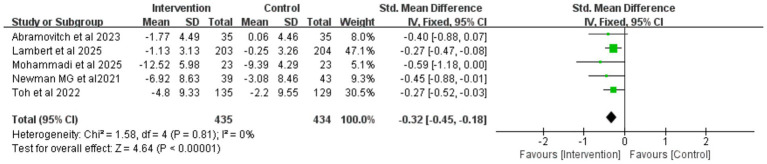
Meta-analysis and forest plot of stress at follow-up.

To evaluate the long-term effects of iCBT intervention, we conducted a meta-analysis of outcome measures during the follow-up period. The results showed that during follow-up, the iCBT intervention group maintained significantly greater improvements compared to the control group in depression (8 studies, *n* = 1,393), anxiety (5 studies, *n* = 887), and stress (5 studies, n = 869), with the following specific results: depression (SMD = −0.28, 95% CI −0.39 to −0.18, *p* < 0.001) (Figure 10), anxiety (SMD = −0.17, 95% CI −0.30 to −0.03, *p* = 0.01) (Figure 11), and stress (SMD = −0.32, 95% CI −0.45 to −0.18, *p* < 0.001) (Figure 12). The effect sizes were small but significant.

## 7 Discussion

### 7.1 Principal findings

Based on a systematic review of 30 RCTs involving 5,169 college students, with 29 studies included in the meta-analysis, iCBT was found to significantly improve symptoms of anxiety, depression, and stress among college students, with sustained intervention effects observed during the follow-up period. Subgroup analyses indicated that intervention format and duration may moderate the effects: chatbot-based interventions appeared to be associated with relatively greater improvements in depression, whereas web platform-based interventions appeared to be more effective in improving anxiety. Additionally, longer intervention durations (>4 weeks) yielded better outcomes than shorter durations. The findings remained robust, with no significant publication bias detected. In conclusion, this systematic review and meta-analysis provide compelling evidence supporting the efficacy of iCBT in improving symptoms of anxiety, depression, and stress among college students.

### 7.2 Comparison to prior work

The findings of this study are consistent with previous meta-analyses on the effectiveness of iCBT across diverse populations. These studies have confirmed that iCBT not only demonstrates positive effects in the general population but also shows significant efficacy in specific patient groups, such as those with cardiovascular diseases or cancer, as well as in adolescents with depression (El-Malahi et al., 2025; Brocklehurst et al., 2024; Shen et al., 2025; Wickersham et al., 2022). In addition to meta-analyses conducted in broader populations, our findings are also generally consistent with evidence synthesis studies focusing on college students, including those by Oliveira et al. (Oliveira et al., 2023), Ledur et al. (Ledur et al., 2023), and Madrid-Cagigal et al. (Madrid-Cagigal et al., 2025). Building on this prior work, the present study further expands and updates the evidence base for iCBT among college students by incorporating depression and stress outcomes, performing subgroup analyses based on intervention format and duration, and quantitatively pooling effects at follow-up. By incorporating more recent studies (a total of 30 RCTs involving 5,169 participants), we have confirmed the comprehensive interventional effects of iCBT across a broader spectrum. To our knowledge, this is the first study to comprehensively evaluate the effects of iCBT on depression, anxiety, and stress in college students while systematically assessing outcomes at follow-up. This further strengthens the evidence base for iCBT as a universal psychological intervention, particularly highlighting its value for high-risk groups such as college students. College students, who are undergoing the transition to early adulthood, often face multiple challenges, including academic pressure, social adaptation, and future planning, which can easily trigger emotional distress. With its advantages of high accessibility, ability to overcome geographical barriers, and reduced stigma, iCBT provides a feasible and promising support pathway for addressing psychological issues in this population.

Regarding the sustainability of intervention effects, this study found that the effects of iCBT remained significant during the follow-up period (depression: SMD = −0.28, *p* < 0.001; anxiety: SMD = −0.17, *p* = 0.01; stress: SMD = −0.32, *p* < 0.001). This finding holds important clinical significance. Previous research on the long-term effects of iCBT has been relatively limited, and this study highlights the potential of iCBT in providing sustainable mental health support. A long-term follow-up study on iCBT for childhood anxiety disorders indicated that its effects can be maintained, and even individuals without complete remission can experience continued improvement through subsequent interventions (Jolstedt et al., 2021). This sustainability is crucial for preventing the recurrence of psychological disorders, particularly considering that adolescence and young adulthood represent the peak periods for the onset of mental disorders, which may lead to lifelong impacts (Stelmach et al., 2022).

The subgroup analysis in this study explored the moderating effects of intervention format and duration on iCBT outcomes. However, due to the limited number of studies reporting stress outcomes, subgroup analysis was not conducted for this measure. Specifically, this study found that chatbot-based interventions may lead to relatively greater improvements in depressive symptoms, whereas web platform-based interventions may be more effective in improving anxiety symptoms. These findings align with existing explorations of different iCBT formats in the field. For instance, the work of Thieme et al. (Thieme et al., 2023), which focuses on human-centered AI in iCBT, demonstrates the potential of AI tools to support clinical practice, resonating with the application direction of chatbots in iCBT. Our subgroup analysis provides preliminary evidence regarding the matching of specific symptoms with intervention formats, suggesting that future iCBT designs could be customized based on target symptoms. For example, chatbots might more effectively address depression-related cognitive biases and emotional dysregulation by providing structured cognitive restructuring exercises and emotional support. In contrast, web platforms, potentially through offering richer resources (such as videos, audio, interactive exercises) and more flexible module combinations, might better address the diverse manifestations of anxiety disorders. Furthermore, this study found that longer iCBT intervention durations (>4 weeks) yielded better outcomes than shorter ones. This aligns with the general pattern observed in many psychological interventions, particularly CBT, where a dosage effect is often positively correlated with intervention outcomes (Durpoix et al., 2024). Longer intervention durations may allow for more adequate skill acquisition, practice, and consolidation, thereby leading to more sustained behavioral changes and symptom improvement. For example, a long-term follow-up study on iCBT for adolescent anxiety disorders showed that sustained intervention could maintain or even further enhance treatment effects (Jolstedt et al., 2021). Notably, a relatively high level of heterogeneity was observed for depression outcomes in this study (*I^2^* = 65%), suggesting substantial variability in college students’ responses to iCBT interventions. This heterogeneity may be attributable to factors such as differences in university settings and cultural backgrounds, baseline symptom severity, variations in intervention formats, and individual levels of digital literacy.

The findings of this study also complement the existing literature on the relationship between anxiety/depression and internet addiction. A complex bidirectional relationship exists between internet addiction and anxiety/depression, meaning that anxiety/depression can lead to internet addiction, and vice versa, potentially creating a vicious cycle (Ismail, 2024). As an effective psychological intervention, iCBT not only directly targets symptoms of anxiety and depression but may also indirectly reduce pathological internet use by addressing these core psychological issues, thereby helping to break this vicious cycle.

### 7.3 Strengths and limitations

This study strictly adhered to the PRISMA reporting guidelines and established a comprehensive and standardized research process. Focusing on randomized controlled trials published from database inception until October 12, 2025, it thoroughly presents the progress in addressing anxiety, depression, and stress among college students. As the first study to systematically review iCBT interventions targeting all three psychological issues—anxiety, depression, and stress—in college students, it further conducted subgroup analyses on intervention type and duration to deeply explore their mechanisms of influence on intervention effects. Additionally, through systematic analysis of follow-up results, it provides crucial empirical support for assessing the long-term sustainability of the intervention effects.

However, this study has several limitations: (1) A certain proportion of the included studies presented moderate to high risks of bias, primarily reflected in issues with allocation concealment and deviations from the intended intervention protocols, which may have influenced the estimation of effect sizes; (2) Some subgroup analyses were based on a limited number of studies (e.g., certain subgroups included only two or three studies), posing a risk of insufficient representativeness. Therefore, the subgroup analyses in this paper should be considered exploratory, and their findings require validation through more high-quality studies in the future; (3) The search scope of this study was limited to RCTs published in English, which may have resulted in the omission of relevant studies in other languages and thus introduced potential language bias. During the initial screening stage, one non-English record was identified; however, it was a systematic review rather than a RCT and was therefore not included in the final analysis. Future research could expand the range of search languages to enhance the comprehensiveness of the evidence and to further examine the cross-cultural applicability of iCBT interventions; (4) Finally, the data on long-term follow-up in the included studies were relatively limited, meaning that the long-term stability of iCBT effects still requires further research for verification.

### 7.4 Implications for future research and recommendations for university mental health practice

#### 7.4.1 Recommendations for future research

Based on the findings and limitations of the present study, future research should conduct more methodologically rigorous RCTs with a lower risk of bias to enhance the overall quality of evidence. It is also necessary to further investigate the mechanisms of action and relative effectiveness of different intervention formats (e.g., chatbots, web-based platforms, and mobile applications), and to clarify their appropriate contexts of use through head-to-head comparative studies.

In addition, given that the present study found interventions lasting longer than 4 weeks to yield more significant effects, future research may optimize intervention duration on this basis and further explore the optimal intervention length for students with varying levels of symptom severity. Incorporating long-term follow-up assessments of 1 year or more would help determine the sustainability of intervention effects and their impact on symptom relapse. Strengthening the reporting of intervention components and adherence may also facilitate more refined subgroup and moderator analyses.

#### 7.4.2 Recommendations for university mental health practice

From a practical perspective, the findings of this study suggest that iCBT can serve as a scalable intervention option within university mental health service systems. When implementing iCBT programs, universities may consider setting an intervention duration of at least 4 weeks, with appropriate adjustments based on the severity of students’ symptoms.

Moreover, given that different intervention formats demonstrate varying effects on anxiety and depression outcomes, universities—where resources permit—may select suitable delivery platforms according to students’ predominant symptom profiles. In addition, establishing phased outcome evaluation and follow-up mechanisms may help enhance the long-term effectiveness and sustainability of iCBT interventions in university settings.

## 8 Conclusion

This systematic review and meta-analysis demonstrate that iCBT is an effective and sustainably beneficial intervention for alleviating symptoms of anxiety, depression, and stress among college students. Subgroup analyses further revealed that intervention effects are influenced by both the format and duration of the intervention: chatbot-based interventions may hold particular advantages in relieving depression, while web platform-based interventions appear more effective in reducing anxiety. Moreover, longer intervention durations (>4 weeks) were associated with significantly better outcomes. Future research should focus on conducting additional high-quality trials and further exploring the long-term effects and cultural adaptability of iCBT.

## Data Availability

The original contributions presented in the study are included in the article/Supplementary material, further inquiries can be directed to the corresponding author.

## Footnotes

1
ClinicalTrials.gov
