# Supplementary material for: The intervention effect of internet-based cognitive behavioral therapy on anxiety, depression, and stress in college students: a systematic review and meta-analysis based on randomized controlled trials
Source: Front Psychol. 2026 Mar 9;17:1745837. doi: 10.3389/fpsyg.2026.1745837 (PMC13006606; doi:10.3389/fpsyg.2026.1745837)
Supplement: Supplementary file 1 [file Table_1.docx]

**Search Strategy:**

**Search strategies for Pubmed**

(((("Cognitive Behavioral Therapy"[Mesh]) OR ((((((((((((((((((((((cognitive behavioral therapy[Title/Abstract]) OR (cognitive behavioral therapies[Title/Abstract])) OR (behavioral therapy, cognitive[Title/Abstract])) OR (therapies, cognitive behavioral[Title/Abstract])) OR (therapy, cognitive behavioral[Title/Abstract])) OR (cognitive behavior therapy[Title/Abstract])) OR (cognitive behavior therapies[Title/Abstract])) OR (therapy, cognitive behavior[Title/Abstract])) OR (therapies, cognitive behavior[Title/Abstract])) OR (cognitive behaviour therapy[Title/Abstract])) OR (cognitive behaviour therapies[Title/Abstract])) OR (therapy, cognitive behaviour[Title/Abstract])) OR (therapies, cognitive behaviour[Title/Abstract])) OR (cognitive therapy[Title/Abstract])) OR (therapy, cognitive[Title/Abstract])) OR (cognition therapies[Title/Abstract])) OR (therapy, cognition[Title/Abstract])) OR (therapies, cognition[Title/Abstract])) OR (cognitive psychotherapy[Title/Abstract])) OR (cognitive psychotherapies[Title/Abstract])) OR (psychotherapy, cognitive[Title/Abstract])) OR (CBT[Title/Abstract]))) AND (((((((((((((((((((((((((((((smartphone[Title/Abstract]) OR (telemedicine[Title/Abstract])) OR (text messaging[Title/Abstract])) OR (telehealth[Title/Abstract])) OR (digital[Title/Abstract])) OR (online therapy[Title/Abstract])) OR (mobile*[Title/Abstract])) OR (mobile phone[Title/Abstract])) OR (mobile app[Title/Abstract])) OR (mobile health[Title/Abstract])) OR (software[Title/Abstract])) OR (mhealth[Title/Abstract])) OR (m-health[Title/Abstract])) OR (ehealth[Title/Abstract])) OR (e-health[Title/Abstract])) OR (electronic health[Title/Abstract])) OR (tele-health[Title/Abstract])) OR (teletherapy[Title/Abstract])) OR (e-mental health[Title/Abstract])) OR (internet-based intervention[Title/Abstract])) OR (internet*[Title/Abstract])) OR (web[Title/Abstract])) OR (web-based[Title/Abstract])) OR (web-delivered[Title/Abstract])) OR (computer*[Title/Abstract])) OR (app[Title/Abstract])) OR (technolog*[Title/Abstract])) OR (virtual reality[Title/Abstract])) OR (VR[Title/Abstract]))) AND (((((university student[Title/Abstract]) OR (university students[Title/Abstract])) OR (college student[Title/Abstract])) OR (college students[Title/Abstract])) OR (undergraduate*[Title/Abstract]))) AND ((("Randomized Controlled Trial" [Publication Type]) OR ("Randomized Controlled Trials as Topic"[Mesh])) OR ((((((randomized controlled trial[Title/Abstract]) OR (randomized[Title/Abstract])) OR (clinical trial[Title/Abstract])) OR (Randomization[Title/Abstract])) OR (Randomisation[Title/Abstract])) OR (RCT[Title/Abstract]))) Sort by: Most Recent 196

**Search strategies for Web of Science**

#1 TS=(smartphone OR telemedicine OR text messaging OR telehealth OR digital OR online therapy OR mobile* OR mobile phone OR mobile app OR mobile health OR software OR mhealth OR m-health OR ehealth OR e-health OR electronic health OR tele-health OR teletherapy OR e-mental health OR internet-based intervention OR internet* OR web OR web-based OR web-delivered OR computer* OR app OR technolog* OR virtual reality OR VR) [3,581,006](http://webofscience.clarivate.cn-s.dres.hactcm.edu.cn/wos/woscc/summary/d04f4bf9-c638-40cb-802c-7c57439bb719-01802e244f/relevance/1)

#2 TS=(university student OR university students OR college student OR college students OR undergraduate*) [137,860](http://webofscience.clarivate.cn-s.dres.hactcm.edu.cn/wos/woscc/summary/89366cbd-3ce6-4181-a0ae-bb4a289effdf-01802e2a04/relevance/1)

#3 TS=(randomized controlled trial OR clinical trial OR randomized OR Randomization OR Randomisation OR RCT) 1,422,377

#4 TS=(cognitive behavioral therapy OR cognitive behavioral therapy OR cognitive behavioral therapies OR cognitive behavior therapy OR cognitive behavior therapies OR cognitive behaviour therapy OR cognitive behaviour therapies OR cognitive therapy OR cognition therapies OR cognitive psychotherapy OR cognitive psychotherapies OR CBT) [84,729](http://webofscience.clarivate.cn-s.dres.hactcm.edu.cn/wos/woscc/summary/0b1a68e8-060b-4732-bb82-41c146b583dd-01802e35dd/relevance/1)

#5 #1 AND #2 AND #3 AND #4 182 12 Oct 2025

**Search strategies for Embase**

#1. 'cognitive behavioral therapy'/exp 45,735

#2. 'cognitive behavioral therapy':ab,ti OR 'cognitive behavioral therapies':ab,ti OR 'behavioral therapy, cognitive':ab,ti OR 'therapies, cognitive behavioral':ab,ti OR 'therapy, cognitive behavioral':ab,ti OR 'cognitive behavior therapy':ab,ti OR 'cognitive behavior therapies':ab,ti OR 'therapy, cognitive behavior':ab,ti OR 'therapies, cognitive behavior':ab,ti OR 'cognitive behaviour therapy':ab,ti OR 'cognitive behaviour therapies':ab,ti OR 'therapy, cognitive behaviour':ab,ti OR 'therapies, cognitive behaviour':ab,ti OR 'cognitive therapy':ab,ti OR 'therapy, cognitive':ab,ti OR 'cognition therapies':ab,ti OR 'therapy, cognition':ab,ti OR 'therapies, cognition':ab,ti OR 'cognitive psychotherapy':ab,ti OR 'cognitive psychotherapies':ab,ti OR 'psychotherapy, cognitive':ab,ti OR CBT:ab,ti 49,448

#3. smartphone:ab,ti OR telemedicine:ab,ti OR 'text messaging':ab,ti OR telehealth:ab,ti OR digital:ab,ti OR 'online therapy':ab,ti OR 'mobile*':ab,ti OR 'mobile phone':ab,ti OR 'mobile app':ab,ti OR 'mobile health':ab,ti OR software:ab,ti OR mhealth:ab,ti OR 'm-health':ab,ti OR ehealth:ab,ti OR 'e-health':ab,ti OR 'electronic health':ab,ti OR 'tele-health':ab,ti OR teletherapy:ab,ti OR 'e-mental health':ab,ti OR 'internet-based intervention':ab,ti OR 'internet*':ab,ti OR web:ab,ti OR 'web-based':ab,ti OR 'web-delivered':ab,ti OR 'computer*':ab,ti OR app:ab,ti OR 'technolog*':ab,ti OR 'virtual reality':ab,ti OR VR:ab,ti 2,756,692

#4. 'university student':ab,ti OR 'university students':ab,ti OR 'college student':ab,ti OR 'college students':ab,ti OR 'undergraduate*':ab,ti 134,103

#5. 'randomized controlled trial':ab,ti OR 'randomized':ab,ti OR 'clinical trial':ab,ti OR 'Randomization':ab,ti OR 'Randomisation':ab,ti OR RCT:ab,ti 1,618,913

#6. #1 OR #2 72,698

#7. #3 AND #4 AND #5 AND #6 177 12 Oct 2025

**Search strategies for Cochrane Library**

#1 MeSH descriptor: [Cognitive Behavioral Therapy] explode all trees 14960

#2 (cognitive behavioral therapy):ti,ab,kw OR (cognitive behavioral therapies):ti,ab,kw OR (behavioral therapy, cognitive):ti,ab,kw OR ( therapies, cognitive behavioral):ti,ab,kw OR ( therapy, cognitive behavioral):ti,ab,kw OR (cognitive behavior therapy):ti,ab,kw OR (cognitive behavior therapies):ti,ab,kw OR (therapy, cognitive behavior ):ti,ab,kw OR (therapies, cognitive behavior):ti,ab,kw OR (cognitive behaviour therapy):ti,ab,kw OR (cognitive behaviour therapies):ti,ab,kw OR (therapy, cognitive behaviour):ti,ab,kw OR (therapies, cognitive behaviour):ti,ab,kw OR (cognitive therapy):ti,ab,kw OR (therapy, cognitive):ti,ab,kw OR (cognition therapies):ti,ab,kw OR (therapy, cognition):ti,ab,kw OR (therapies, cognition):ti,ab,kw OR (cognitive psychotherapy):ti,ab,kw OR (cognitive psychotherapies):ti,ab,kw OR (psychotherapy, cognitive):ti,ab,kw OR (CBT):ti,ab,kw 63551

#3 (smartphone):ti,ab,kw OR (telemedicine):ti,ab,kw OR (text messaging):ti,ab,kw OR (telehealth):ti,ab,kw OR (digital):ti,ab,kw OR (online therapy):ti,ab,kw OR (mobile*):ti,ab,kw OR (mobile phone):ti,ab,kw OR (mobile app):ti,ab,kw OR (mobile health):ti,ab,kw OR (software):ti,ab,kw OR (mhealth):ti,ab,kw OR (m-health):ti,ab,kw OR (ehealth):ti,ab,kw OR (e-health):ti,ab,kw OR (electronic health):ti,ab,kw OR (tele-health):ti,ab,kw OR (teletherapy):ti,ab,kw OR (e-mental health):ti,ab,kw OR (internet-based intervention):ti,ab,kw OR (internet*):ti,ab,kw OR (web):ti,ab,kw OR (web-based):ti,ab,kw OR (web-delivered):ti,ab,kw OR (computer*):ti,ab,kw OR (app):ti,ab,kw OR (technolog*):ti,ab,kw OR (virtual reality):ti,ab,kw OR (VR):ti,ab,kw

219420

#4 (randomized controlled trial):ti,ab,kw OR (randomized):ti,ab,kw OR (clinical trial):ti,ab,kw OR (Randomization):ti,ab,kw OR (Randomisation):ti,ab,kw OR (RCT):ti,ab,kw 1384815

#5 #1 OR #2 65708

#6 (university student):ti,ab,kw OR (university students):ti,ab,kw OR (college student):ti,ab,kw OR (college students):ti,ab,kw OR (undergraduate*):ti,ab,kw 19527

#7 #5 AND #3 AND #4 AND #6 455 12 Oct 2025

**Search strategies for Scopus**

( TITLE-ABS-KEY ("cognitive behavioral therapy" OR "cognitive behavioral therapies" OR "behavioral therapy, cognitive" OR "therapies, cognitive behavioral" OR "therapy, cognitive behavioral" OR "cognitive behavior therapy" OR "cognitive behavior therapies" OR "therapy, cognitive behavior" OR "therapies, cognitive behavior" OR "cognitive behaviour therapy" OR "cognitive behaviour therapies" OR "therapy, cognitive behaviour" OR "therapies, cognitive behaviour" OR "cognitive therapy" OR "therapy, cognitive" OR "cognition therapies" OR "therapy, cognition" OR "therapies, cognition" OR "cognitive psychotherapy" OR "cognitive psychotherapies" OR "psychotherapy, cognitive" OR CBT) AND TITLE-ABS-KEY (smartphone OR telemedicine OR "text messaging" OR telehealth OR digital OR "online therapy" OR mobile* OR "mobile phone" OR "mobile app" OR "mobile health" OR software OR mhealth OR "m-health" OR ehealth OR "e-health" OR "electronic health" OR "tele-health" OR teletherapy OR "e-mental health" OR "internet-based intervention" OR internet* OR web OR "web-based" OR "web-delivered" OR computer* OR app OR technolog* OR "virtual reality" OR VR) AND TITLE-ABS-KEY ("university student" OR "university students" OR "college student" OR "college students" OR undergraduate*) AND TITLE-ABS-KEY ("randomized controlled trial" OR "clinical trial" OR randomized OR Randomization OR Randomisation OR RCT)) 204 12 Oct 2025

**Search strategies for APA PsycInfo (EBSCO)**

S1 [TI (smartphone OR telemedicine OR text messaging OR telehealth OR digital OR online therapy OR mobile* OR mobile phone OR mobile app OR mobile health OR software OR mhealth OR m-health OR ehealth OR e-health OR electronic health OR tele-health OR teletherapy OR e-mental health OR internet-based intervention OR internet* OR web OR web-based OR web-delivered OR computer* OR app OR technolog* OR virtual reality OR VR) OR AB (smartphone OR telemedicine OR text messaging OR telehealth OR digital OR online therapy OR mobile* OR mobile phone OR mobile app OR mobile health OR software OR mhealth OR m-health OR ehealth OR e-health OR electronic health OR tele-health OR teletherapy OR e-mental health OR internet-based intervention OR internet* OR web OR web-based OR web-delivered OR computer* OR app OR technolog* OR virtual reality OR VR)](https://research.ebsco.com/search/results?db=psyh&expanders=concept&limiters=FT:Y&q=TI (smartphone OR telemedicine OR text messaging OR telehealth OR digital OR online therapy OR   mobile* OR mobile phone OR mobile app OR mobile health OR software OR mhealth OR m-health OR   ehealth OR e-health OR electronic health OR tele-health OR teletherapy OR e-mental health OR internet-based intervention OR internet* OR web OR web-based OR web-delivered OR computer* OR app OR technolog*   OR virtual reality OR VR) OR AB (smartphone OR telemedicine OR text messaging OR telehealth OR digital OR online therapy OR   mobile* OR mobile phone OR mobile app OR mobile health OR software OR mhealth OR m-health OR   ehealth OR e-health OR electronic health OR tele-health OR teletherapy OR e-mental health OR internet-based intervention OR internet* OR web OR web-based OR web-delivered OR computer* OR app OR technolog*   OR virtual reality OR VR)&qm=W3sidmFsdWUiOiJzbWFydHBob25lIE9SIHRlbGVtZWRpY2luZSBPUiB0ZXh0IG1lc3NhZ2luZyBPUiB0ZWxlaGVhbHRoIE9SIGRpZ2l0YWwgT1Igb25saW5lIHRoZXJhcHkgT1IgICBtb2JpbGUqIE9SIG1vYmlsZSBwaG9uZSBPUiBtb2JpbGUgYXBwIE9SIG1vYmlsZSBoZWFsdGggT1Igc29mdHdhcmUgT1IgbWhlYWx0aCBPUiBtLWhlYWx0aCBPUiAgIGVoZWFsdGggT1IgZS1oZWFsdGggT1IgZWxlY3Ryb25pYyBoZWFsdGggT1IgdGVsZS1oZWFsdGggT1IgdGVsZXRoZXJhcHkgT1IgZS1tZW50YWwgaGVhbHRoIE9SIGludGVybmV0LWJhc2VkIGludGVydmVudGlvbiBPUiBpbnRlcm5ldCogT1Igd2ViIE9SIHdlYi1iYXNlZCBPUiB3ZWItZGVsaXZlcmVkIE9SIGNvbXB1dGVyKiBPUiBhcHAgT1IgdGVjaG5vbG9nKiAgIE9SIHZpcnR1YWwgcmVhbGl0eSBPUiBWUiIsInR5cGUiOiJmaWVsZCIsImNvZGUiOiJUSSJ9LHsidmFsdWUiOiJPUiIsInR5cGUiOiJsb2dpYyJ9LHsidmFsdWUiOiJzbWFydHBob25lIE9SIHRlbGVtZWRpY2luZSBPUiB0ZXh0IG1lc3NhZ2luZyBPUiB0ZWxlaGVhbHRoIE9SIGRpZ2l0YWwgT1Igb25saW5lIHRoZXJhcHkgT1IgICBtb2JpbGUqIE9SIG1vYmlsZSBwaG9uZSBPUiBtb2JpbGUgYXBwIE9SIG1vYmlsZSBoZWFsdGggT1Igc29mdHdhcmUgT1IgbWhlYWx0aCBPUiBtLWhlYWx0aCBPUiAgIGVoZWFsdGggT1IgZS1oZWFsdGggT1IgZWxlY3Ryb25pYyBoZWFsdGggT1IgdGVsZS1oZWFsdGggT1IgdGVsZXRoZXJhcHkgT1IgZS1tZW50YWwgaGVhbHRoIE9SIGludGVybmV0LWJhc2VkIGludGVydmVudGlvbiBPUiBpbnRlcm5ldCogT1Igd2ViIE9SIHdlYi1iYXNlZCBPUiB3ZWItZGVsaXZlcmVkIE9SIGNvbXB1dGVyKiBPUiBhcHAgT1IgdGVjaG5vbG9nKiAgIE9SIHZpcnR1YWwgcmVhbGl0eSBPUiBWUiIsInR5cGUiOiJmaWVsZCIsImNvZGUiOiJBQiJ9XQ==&searchMode=all&sort=relevance&userDirectAction=true) 197,079

S2 TI (university student OR university students OR college student OR college students OR undergraduate*) OR AB (university student OR university students OR college student OR college students OR undergraduate*) 141,541

S3 TI (randomized controlled trial OR clinical trial OR randomized OR Randomization OR Randomisation OR RCT) OR AB (randomized controlled trial OR clinical trial OR randomized OR Randomization OR Randomisation OR RCT) 66,329

S4 [TI (cognitive behavioral therapy OR cognitive behavioral therapy OR cognitive behavioral therapies OR cognitive behavior therapy OR cognitive behavior therapies OR cognitive behaviour therapy OR cognitive behaviour therapies OR cognitive therapy OR cognition therapies OR cognitive psychotherapy OR cognitive psychotherapies OR CBT) OR AB (cognitive behavioral therapy OR cognitive behavioral therapy OR cognitive behavioral therapies OR cognitive behavior therapy OR cognitive behavior therapies OR cognitive behaviour therapy OR cognitive behaviour therapies OR cognitive therapy OR cognition therapies OR cognitive psychotherapy OR cognitive psychotherapies OR CBT)](https://research.ebsco.com/search/results?db=psyh&expanders=concept&limiters=FT:Y&q=TI (cognitive behavioral therapy OR cognitive behavioral therapy OR cognitive behavioral therapies OR cognitive behavior therapy OR cognitive behavior therapies OR cognitive behaviour therapy OR cognitive behaviour therapies OR cognitive therapy OR cognition therapies OR cognitive psychotherapy OR cognitive psychotherapies OR CBT) OR AB (cognitive behavioral therapy OR cognitive behavioral therapy OR cognitive behavioral therapies OR cognitive behavior therapy OR cognitive behavior therapies OR cognitive behaviour therapy OR cognitive behaviour therapies OR cognitive therapy OR cognition therapies OR cognitive psychotherapy OR cognitive psychotherapies OR CBT)&qm=W3sidmFsdWUiOiJjb2duaXRpdmUgYmVoYXZpb3JhbCB0aGVyYXB5IE9SIGNvZ25pdGl2ZSBiZWhhdmlvcmFsIHRoZXJhcHkgT1IgY29nbml0aXZlIGJlaGF2aW9yYWwgdGhlcmFwaWVzIE9SIGNvZ25pdGl2ZSBiZWhhdmlvciB0aGVyYXB5IE9SIGNvZ25pdGl2ZSBiZWhhdmlvciB0aGVyYXBpZXMgT1IgY29nbml0aXZlIGJlaGF2aW91ciB0aGVyYXB5IE9SIGNvZ25pdGl2ZSBiZWhhdmlvdXIgdGhlcmFwaWVzIE9SIGNvZ25pdGl2ZSB0aGVyYXB5IE9SIGNvZ25pdGlvbiB0aGVyYXBpZXMgT1IgY29nbml0aXZlIHBzeWNob3RoZXJhcHkgT1IgY29nbml0aXZlIHBzeWNob3RoZXJhcGllcyBPUiBDQlQiLCJ0eXBlIjoiZmllbGQiLCJjb2RlIjoiVEkifSx7InZhbHVlIjoiT1IiLCJ0eXBlIjoibG9naWMifSx7InZhbHVlIjoiY29nbml0aXZlIGJlaGF2aW9yYWwgdGhlcmFweSBPUiBjb2duaXRpdmUgYmVoYXZpb3JhbCB0aGVyYXB5IE9SIGNvZ25pdGl2ZSBiZWhhdmlvcmFsIHRoZXJhcGllcyBPUiBjb2duaXRpdmUgYmVoYXZpb3IgdGhlcmFweSBPUiBjb2duaXRpdmUgYmVoYXZpb3IgdGhlcmFwaWVzIE9SIGNvZ25pdGl2ZSBiZWhhdmlvdXIgdGhlcmFweSBPUiBjb2duaXRpdmUgYmVoYXZpb3VyIHRoZXJhcGllcyBPUiBjb2duaXRpdmUgdGhlcmFweSBPUiBjb2duaXRpb24gdGhlcmFwaWVzIE9SIGNvZ25pdGl2ZSBwc3ljaG90aGVyYXB5IE9SIGNvZ25pdGl2ZSBwc3ljaG90aGVyYXBpZXMgT1IgQ0JUIiwidHlwZSI6ImZpZWxkIiwiY29kZSI6IkFCIn1d&searchMode=all&sort=relevance&userDirectAction=true) 28,963

S5 [S4 AND S1 AND S2 AND S3](https://research.ebsco.com/search/results?combinedSearchQueryId=sq:3da46280-efb2-4f44-8e5a-52897fcf0e6b&db=psyh&expanders=concept&limiters=FT:Y&q=S4 AND S1 AND S2 AND S3&searchMode=all&sort=relevance&userDirectAction=true) 54 12 Oct 2025

**Search strategies for PsychARTICLES (EBSCO)**

The retrieval steps are the same as those for APA PsycInfo. 3 12 Oct 2025

**Search strategies for CINAHL (EBSCO)**

The retrieval steps are the same as those for APA PsycInfo. 44 12 Oct 2025

**Search strategies for ClinicalTrials.**

Intervention/treatment: (cognitive behavioral therapy OR cognitive behavioral therapy OR cognitive behavioral therapies OR cognitive behavior therapy OR cognitive behavior therapies OR cognitive behaviour therapy OR cognitive behaviour therapies OR cognitive therapy OR cognition therapies OR cognitive psychotherapy OR cognitive psychotherapies OR CBT) AND (smartphone OR telemedicine OR text messaging OR telehealth OR digital OR online therapy OR mobile* OR mobile phone OR mobile app OR mobile health OR software OR mhealth OR m-health OR ehealth OR e-health OR electronic health OR tele-health OR teletherapy OR e-mental health OR internet-based intervention OR internet* OR web OR web-based OR web-delivered OR computer* OR app OR technolog* OR virtual reality OR VR)

Title and/or Title Acronym: university student OR university students OR college student OR college students OR undergraduate* 63 12 Oct 2025
