# Supplementary material for: The intervention effect of internet-based cognitive behavioral therapy on anxiety, depression, and stress in college students: a systematic review and meta-analysis based on randomized controlled trials
Source: Front Psychol. 2026 Mar 9;17:1745837. doi: 10.3389/fpsyg.2026.1745837 (PMC13006606; doi:10.3389/fpsyg.2026.1745837)

Multimedia Appendix 3

****The Intervention Effect of Internet-Based Cognitive Behavioral Therapy on Anxiety, Depression, and Stress in College Students: A Systematic Review and Meta-Analysis Based on Randomized Controlled Trials****

**[Funnel plot](#_Toc4095)** [1](#_Toc4095)

**[Figure S1](#_Toc25828)**[. depression 1](#_Toc25828)

**[Figure S2](#_Toc7289)**[. anxiety 1](#_Toc7289)

**[Subgroup analyses](#_Toc4634)**[: Different types of interventions 2](#_Toc4634)

**[Figure S3](#_Toc24206)**[. depression 2](#_Toc24206)

**[Figure S4](#_Toc10717)**[. anxiety 2](#_Toc10717)

**[Subgroup analyses](#_Toc19417)**[: Duration of intervention 3](#_Toc19417)

**[Figure S5](#_Toc22617)**[. depression 3](#_Toc22617)

**[Figure S6](#_Toc8432)**[. anxiety 3](#_Toc8432)

**Funnel plot**

**Figure S1**. Funnel plot of depression


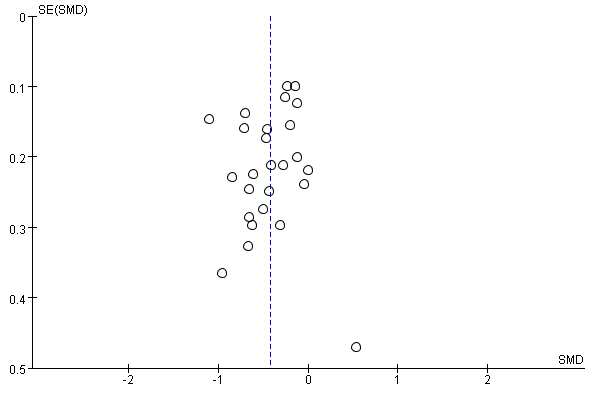


**Figure S2**. Funnel plot of anxiety


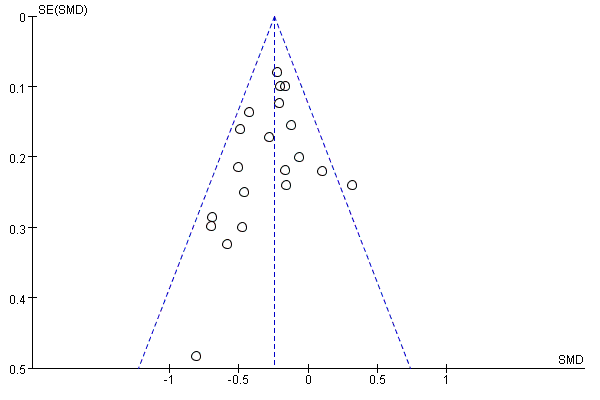


**Subgroup analyses:** Results of Subgroup Analysis Based on Intervention Type (APP, Web-based Platform, and Chatbot)

**Figure S3**. depression

**
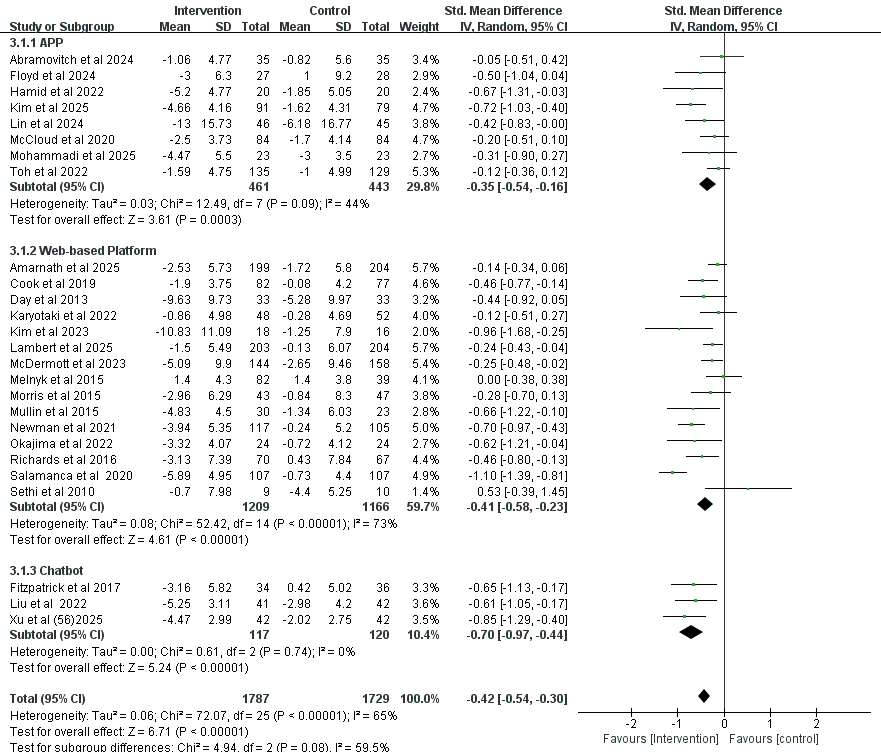
**

**Figure S4**. anxiety

**
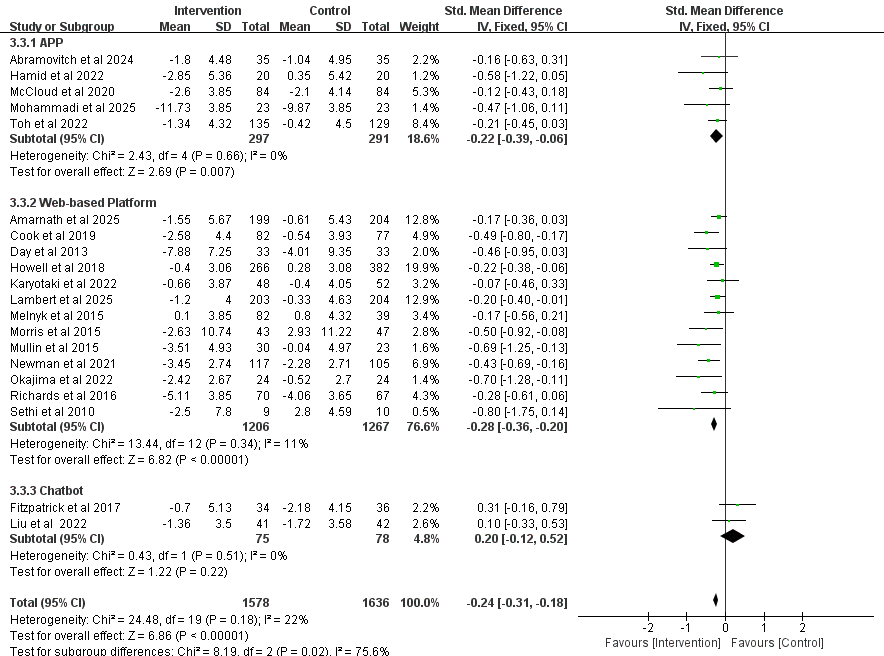
**

**Subgroup analyses:** Subgroup Analysis Based on Intervention Duration (≤4 weeks, >4 weeks and ≤8 weeks, >8 weeks)

**Figure S5**. depression


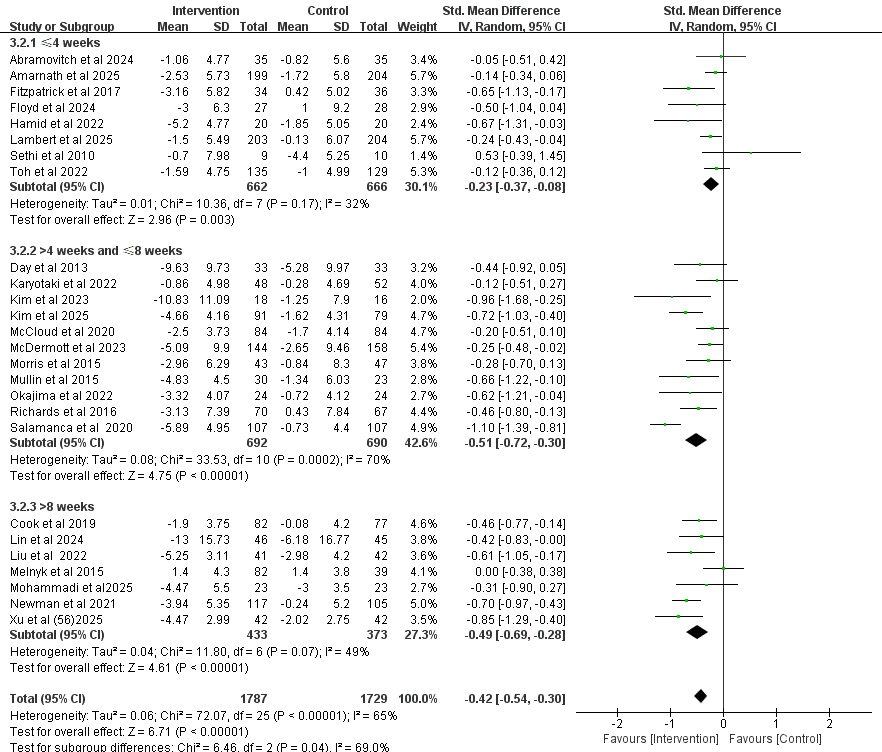


**Figure S6**. anxiety


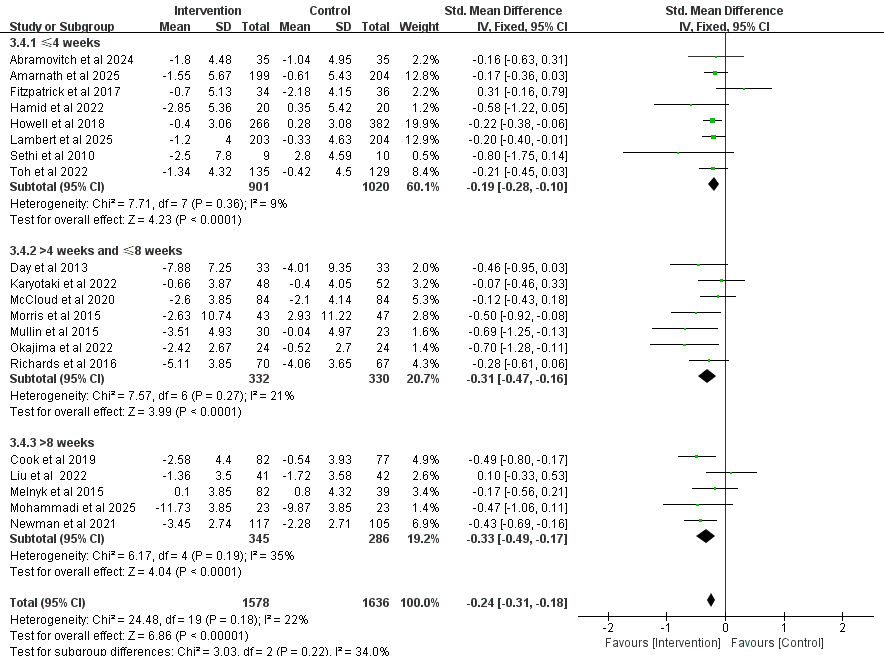

Supplement: Supplementary file 2 [file Table_2.docx]
